# MARKERS OF COGNITIVE RESERVE AND DEMENTIA INCIDENCE IN THE ENGLISH LONGITUDINAL STUDY OF AGEING

**DOI:** 10.1093/geroni/igz038.2310

**Published:** 2019-11-08

**Authors:** Pamela Almeida-Meza, Pamela Almeida-Meza, Andrew Steptoe, Dorina Cadar

**Affiliations:** 1 University College London, London, United Kingdom; 2 Behavioural Science and Health, University College London, London, England, United Kingdom

## Abstract

We investigated a multi-faceted index of cognitive reserve (CR), in relation to dementia incidence over 15 years follow-up in a representative sample of the English population. Data were 12,293 participants aged 50+ from the English Longitudinal Study of Ageing, dementia-free at baseline. CR was derived as a composite measure of education, occupation and leisure activities using a standardised questionnaire. From the overall sample, 603 participants developed dementia. Higher CR levels were associated with lower dementia risk (medium CR: HR 0.72, 95% CI 0.59-0.88, p=0.002 and high CR: HR 0.73, 95% CI 0.58-0.90, p=0.005) compared with lowest levels. These associations were independent of sex, marital status, wealth, smoking, depressive symptoms and poor physical health. Further individual analyses of CR sub-components showed that leisure activities (HR 0.72, 95% CI 0.56-0.91, p=0.007) were linked with reduced dementia risk, contributing to a higher CR and increased overall mental resilience.

